# Validation of SenseWear Armband and ActiHeart monitors for assessments of daily energy expenditure in free-living women with chronic obstructive pulmonary disease

**DOI:** 10.1002/phy2.150

**Published:** 2013-11-26

**Authors:** Nighat Farooqi, Frode Slinde, Lena Håglin, Thomas Sandström

**Affiliations:** 1 Department of Public Health and Clinical Medicine, Respiratory Medicine and Allergy, Umeå UniversityUmeå, Sweden; 2 Department of Internal Medicine and Clinical Nutrition, Sahlgrenska Academy, University of GothenburgGothenburg, Sweden; 3 Department of Public Health and Clinical Medicine, Family Medicine, Umeå UniversityUmeå, Sweden

**Keywords:** Energy expenditure, validity of ActiHeart, validity of SenseWear Armband, women with COPD

## Abstract

To provide individually adapted nutritional support to patients with chronic obstructive pulmonary disease (COPD), objective and reliable methods must be used to assess patient energy requirements. The aim of this study was to validate the use of SenseWear Armband (SWA) and ActiHeart (AH) monitors for assessing total daily energy expenditure (TEE) and activity energy expenditure (AEE) and compare these techniques with the doubly labeled water (DLW) method in free-living women with COPD. TEE and AEE were measured in 19 women with COPD for 14 days using SWAs with software version 5.1 (TEE_SWA5_, AEE_SWA5_) or 6.1 (TEE_SWA6_, AEE_SWA6_) and AH monitors (TEE_AH_, AEE_AH_), using DLW (TEE_DLW_) as the criterion method. The three methods were compared using intraclass correlation coefficient (ICC) and Bland–Altman analyses. The mean TEE did not significantly differ between the DLW and SWA5.1 methods (−21 ± 726 kJ/day; *P* = 0.9), but it did significantly differ between the DLW and SWA6.1 (709 ± 667 kJ/day) (*P* < 0.001) and the DLW and AH methods (709 ± 786 kJ/day) (*P* < 0.001). Strong agreement was observed between the DLW and TEE_SWA5_ methods (ICC = 0.76; 95% CI 0.47–0.90), with moderate agreements between the DLW and TEE_SWA6_ (ICC = 0.66; 95% CI 0.02–0.88) and the DLW and TEE_AH_ methods (ICC = 0.61; 95% CI 0.05–0.85). Compared with the DLW method, the SWA5.1 underestimated AEE by 12% (*P* = 0.03), whereas the SWA6.1 and AH monitors underestimated AEE by 35% (*P* < 0.001). Bland–Altman plots revealed no systematic bias for TEE or AEE. The SWA5.1 can reliably assess TEE in women with COPD. However, the SWA6.1 and AH monitors underestimate TEE. The SWA and AH monitors underestimate AEE.

## Introduction

The impact of chronic obstructive pulmonary disease (COPD) is significantly understudied in women, but current evidence suggests substantial gender differences in the susceptibility to, severity of, and response to COPD management (Varkey 2004). In Sweden, the prevalence of COPD has increased more among women than among men, likely as a result of long-term smoking (Löfdahl and Ström 2007), and this disease has emerged as an important women's health issue.

Bodyweight (BW) loss and/or low fat-free mass (FFM) are common in COPD patients and have been associated with reduced performance and increased morbidity and mortality (Schols et al. 2005; Slinde et al. 2005; King et al. 2008). Nutritional intervention studies in COPD patients have revealed beneficial effects of good nutrition on BW, muscle strength, body composition, respiratory function, and survival (Aniwidyaningsih et al. 2008; King et al. 2008; Farooqi et al. 2011; Collins et al. 2012). It is therefore important to identify COPD patients who are at risk of low BW and/or FFM to optimize their diet. To maintain an energy balance, it is essential to accurately quantify total energy requirements (Goris et al. 2003). Nutritional support to maintain an energy balance in chronic disease patients with malnutrition is often based on equations that predict resting metabolic rates (RMRs) and the inclusion of theoretical factors covering disease-specific effects on energy requirements and physical activity (Reeves and Capra 2003). However, this method can result in inaccurate assessments of energy requirements (Flancbaum et al. 1999; MacDonald and Hildebrandt 2003),especially in patients with COPD because of large individual variations in total daily energy expenditure (TEE) and physical activity levels (PALs) in this group (Slinde et al. 2003; Watz et al. 2009; Troosters et al. 2010; Rabinovich et al. 2013). Thus, objective methods are desirable for assessing TEE in these individuals because individualized nutritional treatment is considered important (Fernandes and Bezerra 2006).

The doubly labeled water (DLW) method is considered the “gold standard” for TEE assessments. DLW has been used to measure TEE in studies of COPD in populations consisting of both genders, but it has never been used in a study that focused exclusively on women with COPD (Baarends et al. 1997; Slinde et al. 2003). The high cost of DLW and the significant technical expertise required for the implementation and analysis of DLW complicate its use in daily clinical settings. Therefore, less expensive and more practical objective methods are required to measure TEE. Several motion sensors have been used to assess TEE and activity energy expenditure (AEE) in COPD patients (Pitta et al. 2006; Patel et al. 2007; Cavalheri et al. 2011; Rabinovich et al. 2013), but few of these assessments were conducted in free-living individuals. To date, no studies evaluating the validity of motion sensors have been performed in women with COPD.

The SenseWear®Pro 2 Armband (SWA; BodyMedia Inc., Pittsburgh, PA) is a portable, multisensor biaxial device worn on the upper right arm that estimates TEE and AEE. It has been used to measure AEE in COPD patients (Watz et al. 2009; Troosters et al. 2010; Waschki et al. 2012; Rabinovich et al. 2013) in free-living state, however, for measuring TEE, it has primarily been used in laboratory settings (Patel et al. 2007; Cavalheri et al. 2011; Hill et al. 2010).The ActiHeart (AH; Cambridge Neurotechnology Ltd., Papworth, UK) monitor is a single-piece combined heart rate and movement monitor designed to clip onto two standard ECG chest electrodes. The AH monitor provides estimates of TEE, RMR, and AEE, but has not yet been validated in COPD patients. Given the growing need to provide individually adapted nutritional support to COPD patients who exhibit a negative energy balance, it is important to have validated, inexpensive, and simple to administer methods.

The aim of this study was to validate two monitors, the SenseWear®Pro 2 Armband and the AH monitor, for TEE assessment in free-living women with COPD. The DLW method was used as a criterion method. The secondary aim was to evaluate the accuracy of the AEE estimates obtained using the SWA and AH monitors by comparing them to the DLW method.

## Material and Methods

### Study design and protocol

This validation study was conducted in the Department of Medicine, the Respiratory Medicine and Allergy Unit, and the Clinical Research Center at Umeå University Hospital in Umeå. Each study patient was instructed not to engage in any rigorous physical activity and not to eat after 11 pm on the day prior to the testing day. At the hospital, a blood sample was collected from each patient for measurements of arterial oxygen tension (Po_2_) and arterial carbon dioxide tension (Pco_2_).The BW, height, and RMR of each patient were also measured. Following the RMR measurements, breakfast was served. In total, each study visit lasted 5–6 h, and the total study duration was 14 days for each participant. Pulmonary function tests were conducted within the first 3 months following the initial 14-day study period.

TEE was assessed over 14 days by DLW analysis. TEE was also simultaneously assessed using two different monitors (the SenseWear® Armband and the AH monitor). A home visit was performed between days 8–10 of the study. The purpose of the home visit was to download data, charge or change the batteries in each of the two monitors, and ensure the appropriate collection and storage of urine samples.

The Ethical Review Board of Umeå University approved the study, and written informed consent was obtained from each of the patients prior to their participation in the study.

### Subjects

Nineteen women with COPD attending the outpatient clinic in the Department of Medicine, Respiratory Medicine and Allergy Unit at Umeå University Hospital in Umeå, were identified and recruited if they fulfilled the inclusion criteria.

#### Inclusion criteria

Patients with clinically stable COPD (Global Initiative for Chronic Obstructive Pulmonary Disease [GOLD], 2009: http://www.goldcopd.org., 12 February 2009], Stage II–III) and a body mass index (BMI) between 18.5 and 30.0 kg/m^2^ (http://www.who.int., World Health Organization 2009) were selected. Body weights and heights were measured, and BMI was calculated for each patient prior to her inclusion in the study.

#### Exclusion criteria

Patients with a history of oxygen therapy, insulin-treated diabetes, thyroid dysfunction, or myopathic or neoplastic disease were excluded from the study.

### Anthropometry

Bodyweight and height were measured at the start of the study, and BW was measured again at the end of the 14-day study period. The weight history of each patient for the past 6 months was also obtained. BW was measured to the nearest 0.1 kg using a digital scale while the participant was wearing light clothing. The height of each participant was measured to the nearest 0.5 cm using a horizontal headboard with an attached wall-mounted metric scale. BMI was then calculated based on these measurements (BW in kg/height in m^2^).

### Pulmonary function measurements

Dynamic and static pulmonary function tests were performed (Jaeger, MasterScreen Body and MasterScreen PFT; CareFusion, Höchberg, Germany). Oxygen saturation was measured during periods of rest and activity using a pulse oximeter. An automatic analyzer was used to measure arterial Po_2_ and Pco_2_ in samples of arterial blood that were drawn from the radial artery using a syringe.

### Total daily energy expenditure

#### DLW method

The DLW method involves administering a dose of stable isotopes of deuterium (^2^H) and oxygen (^18^O) and subsequently measuring the rates of elimination of these isotopes from the body over time. A urine sample was collected from each patient for determinations of background isotope enrichment prior to ingestion of DLW on day 1. Each patient then ingested a weighed mixture of deuterated water that was enriched with ^18^O (0.05 g deuterium oxide [^2^H_2_O] and 0.10 g ^18^O-containing water [^18^O] per kg of body weight). DLW was served in plastic glasses that were weighed before being filled with DLW and again following ingestion to obtain the exact dose of DLW that was ingested. Each patient consumed a glass of tap water after ingesting the DLW, and the exact time of dosing was recorded. Each patient was provided with 21 screw-capped labeled glass vials to fill with urine samples on days 2, 3, 4, 8, 13, 14, and 15. Patients were instructed not to use the first voiding of the day for urine samples. The exact voiding times were registered, and the urine samples were stored in a freezer prior to being delivered to the laboratory. Urine samples were analyzed in triplicate using a Finnigan MAT Delta Plus Isotope-Ratio Mass Spectrometer (ThermoFinnigan, Uppsala, Sweden). TEE (kJ/day) was calculated using the multipoint method by linear regression based on the difference between the elimination constants of deuterium and oxygen-18, with the assumption of fractionation. The relationship between pool size deuterium (N_D_) and pool size oxygen-18 (N_O_) was used as a quality measurement. The acceptable range of this relationship (N_D_/N_O_) has been proposed by the IAEA to be between 1.015 and 1.060 (IAEA 1990).The respiratory quotient was set at 0.85 for calculations of the energy equivalence of CO_2_ produced (Black et al. 1986).

#### SenseWear®Pro 2 Armband

The SenseWear®Pro 2 Armband (BodyMedia Inc.) is a portable, multisensor body monitor that integrates a biaxial accelerometer (longitudinal and transverse) and physiological sensors (heat flux, galvanic skin response, skin temperature, and near-body temperature).This monitor can be used to calculate the TEE and AEE of individuals for whom the body weight, height, handedness, and smoking status are known. The device is worn on the upper portion (triceps) of the right arm. Each patient received a SWA at the time of their first visit. They were instructed to wear the armband at all times, except during bathing, both day and night. Thorough oral and written instructions regarding the appropriate use of SWAs were provided. The patients were also instructed to keep a record of the times that they were not wearing this device, including a list of the activities performed during those periods. The data were sampled in 1-min intervals and were used in combination with each patient's characteristics to estimate TEE using proprietary algorithms. Data were evaluated using InnerView Professional software, versions 5.1 and 6.1 (BodyMedia Inc.).

#### ActiHeart

The AH (Cambridge Neurotechnology Ltd., Papworth, UK) monitor is a heart rate recorder with an integrated accelerometer that is capable of storing time-sequenced data. This monitor is worn on the chest with two ECG electrodes, and it can be used to calculate TEE, RMR, AEE, diet-induced thermo genesis, and PAL based on the user's age, gender, weight, and height (Brage et al. 2005). All patients were given an AH at the time of their first visit. Prior to performing long-term recordings of the patients, a signal test and a step test were performed in accordance with the manufacturer's instructions to ensure the correct placement of the device and to synchronize the AH at an individual level. The step test is an 8-min program that starts at a low pace and increases in intensity over time and in this study none of the patients could complete the whole step test. The data were sampled in 1-min intervals and were used in combination with each patient's characteristics to estimate TEE and AEE using proprietary algorithms. Patients were instructed to wear the AH at all times, including at night. However, wearing this device while bathing was optional. Thorough oral and written instructions regarding the appropriate use of the AH were provided to each patient. All patients were also instructed to keep a journal documenting the periods during which they were not wearing the AH and the activities that were performed during those periods.

### Resting metabolic rate

The RMR was measured by indirect calorimetry (Deltatrac™ II Metabolic Monitor, Datex, Helsinki, Finland) using a ventilated hoodsystem. Patients arrived on the test day in a fasting state, and the RMR of each patient was measured for 30 min after the patients had rested in a supine position for 30 min. All patients were awake during measurement collections. Prior to each measurement, the equipment was calibrated with gas mixtures (O_2_ and CO_2_) according to the manufacturer's instructions. All measurements were performed at a room temperature of 22–24°C. The results shown here are based on the last 25 min of measurement.

### Activity energy expenditure

For the purposes of the current study, AEE was defined as the energy that an individual expends during all of the movements that were performed during the daily study period. The criterion values for AEE were calculated as TEE–RMR, which were measured using the DLW method (TEE) and indirect calorimetry (RMR).

#### Method 1

Activity energy expenditure was estimated from SWA as TEE–RMR, as measured using SWA and the Harris–Benedict equation (Harris and Benedict 1919). Because the SWA assesses TEE, but not RMR, we used the Harris–Benedict equation to assess RMR in women: 655.1 + (9.563 × weight in kg) + (1.850 × height in cm) − (4.676 × age in years). AEE was also calculated from AH as TEE − RMR, which was measured from AH. Thus, AH provides estimations of both TEE and RMR.

#### Method 2

AEE was also estimated from SWA (TEE_SWA_ − RMR) and AH (TEE_AH_ − RMR) using RMR measured by indirect calorimetry. Thus, RMR from indirect calorimetry was used in all the calculations of AEE.

### Smoking habits

Information about smoking habits was collected through interviews and was recorded as the number of pack years.

### Statistical analysis

TEE and AEE estimates from the SWA, software version 5.1 and 6.1 (TEE_SWA5_, AEE_SWA5_, TEE_SWA6_, and AEE_SWA6_, in kJ/day), and AH (TEE_AH_ and AEE_AH_, in kJ/day) were calculated as the mean value obtained over the 14-day measurement period. These values were compared with the mean TEE and AEE values that were obtained using the DLW method (TEE_DLW_ and AEE_DLW_, in kJ/day). To be included in the present analysis, each patient had to wear the SWA and AH for at least 22 h/day and at least for 12 days. When data for any day of the 14-day measurement period were missing, the average TEE and AEE values obtained on the measured days were used in the analysis.

The data were analyzed using the statistical program SPSS version 19.0 (Statistical Package for the Social Sciences; SPSS Inc., Chicago, IL). Descriptive statistics, such as means, standard deviations, and minimum and maximum values, were used. To assess differences between the mean TEE and AEE estimates obtained by the SWA, AH, and DLW methods, we used paired *t*-test. To determine the strength of the relationship between the TEE and AEE estimates that were obtained using the SWA, AH, and DLW methods, we calculated Pearson's correlation coefficient (Cohen 1988). To examine the degree of agreement between estimates of TEE and AEE using the SWA, AH, and DLW methods, we calculated intraclass correlation coefficients (ICCs) assuming a two-way analysis of variance. For these correlation analyses, the closer the correlation is to 1.0, the lower the within-subject variance and the greater the concordance between the estimates (Landis and Koch 1977). Bland–Altman plots were constructed to examine the degree of systematic bias (i.e., if there is any relationship between the magnitude of energy expenditure and differences between the methods) and to calculate the limits of agreement between the monitors and the criterion method, DLW. The level of significance was set at 0.05.

## Results

The general patient characteristics are shown in Table 1. Thirteen women (68%) had quit smoking, and six were current smokers. Seven women had a BMI > 25 kg/m^2^. The BW did not change in any of the patients during the study period.

**Table 1:** General characteristic of women with COPD

	*N*	Mean ± SD	Min–max
Age, years	19	69.2 ± 6.0	59.7–80.0
Weight, kg	19	63.5 ± 10.7	46.8–88.0
BMI, kg/m^2^	19	24.5 ± 3.5	18.5–30.0
% IBW	19	99.0 ± 14.0	75–124
No. pack years	19	27.7 ± 9.0	14–42
Arterial Pco_2_, kPa	16	5.2 ± 0.6	4.3–6.7
Arterial Po_2_, kPa	16	10.2 ± 2.9	4.4–18.4
FEV/FVC, liters	16	0.43 ± 0.12	0.24–0.65
FEV1, % predicted value	16	56.0 ± 15.0	30–78
DLCO, % predicted value	16	47.0 ± 13.0	28–71
IC, % predicted value	16	95.0 ± 20.0	60–134

BMI, body mass index; % IBW, percent of reference weight; FEV, forced expiratory volume; FVC, forced vital capacity; FEV1, forced expiratory volume in 1 sec; DLCO, diffusing capacity of the lung for carbon monoxide; IC, inspiratory capacity.

Pulmonary function tests were performed within the 3 months following the 14-day study period in 16 of the women who participated in the study. Of the three remaining patients, one died of heart failure, one moved to southern Sweden, and one refused to participate.

### Total daily energy expenditure

Total daily energy expenditure, as measured by the DLW, SWA, and AH methods, and RMR, as measured by indirect calorimetry, were obtained for all 19 patients. The overall compliance in wearing the SWA and AH monitor was excellent. No long-term periods of failing to wear these monitors were detected from the diaries or upon cross-checking the diaries with the readings from the monitors. The average ± SD measured wear time for the SWA was 98 ± 3%. No large gaps (>15 min) were recorded, and the brief gaps in the data were autofilled. Similarly, only brief pauses in AH wearing time were recorded, and these periods were also autofilled. Two women had registered SWA and AH data for only 13 days; therefore, an average of 13 days was used for all of the analyses. The measured N_D_/N_O_ values obtained using the DLW method were between 1.018 and 1.048.

**Figure 1:**
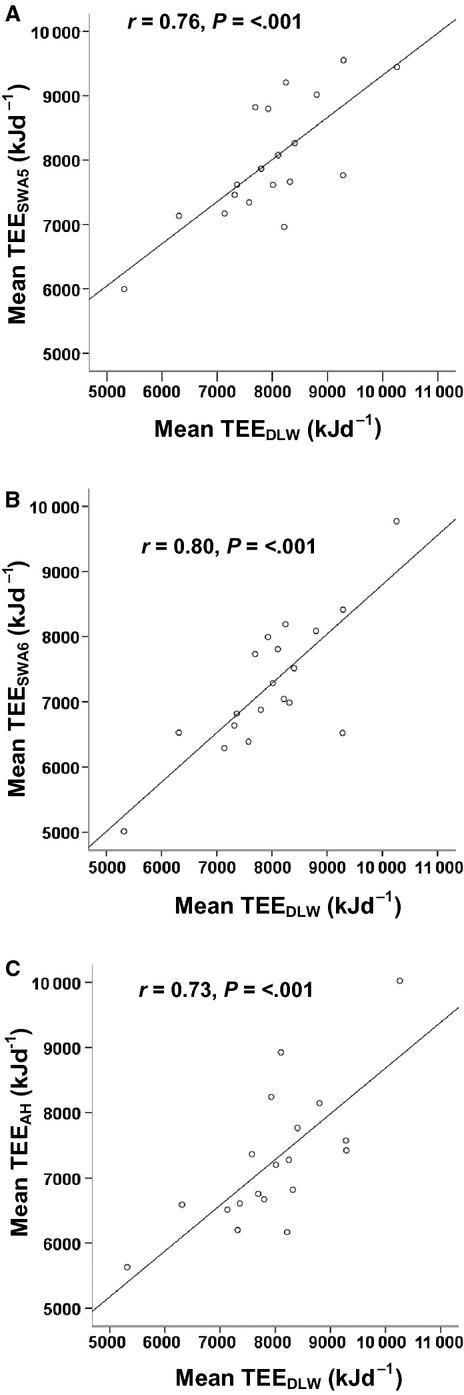
Pearson's correlations between the mean TEE measured by the DLW method (TEE_DLW_) and that measured by (A) the SenseWear Armband, version 5.1 (TEE_SWA5_), (B) the SenseWear Armband, version 6.1 (TEE_SWA6_), and (C) the ActiHeart (TEE_AH_) method in 19 women with COPD. TEE, total energy expenditure; DLW, doubly labeled water.

Table 2 shows the TEE estimates obtained using the DLW, SWA, and AH methods and the RMR values that were measured using indirect calorimetry. A considerable amount of variation was observed in both RMR and TEE (as measured by DLW, SWA version 5.1 and 6.1 and AH) within this COPD group. Paired *t*-tests revealed a mean difference ± SD of −21 ± 726 kJ/day (*P* = 0.9) between TEE_DLW_ and TEE_SWA5_, 709 ± 667 kJ/day (*P* = 0.001) between TEE_DLW_ and TEE_SWA6_, and 709 ± 786 kJ/day (*P* < 0.001) between TEE_DLW_ and TEE_AH._ Assessments of TEE using SWA 5.1 showed an overestimation by 0.3% compared with the result obtained using the criterion method, whereas both the SWA 6.1 and AH methods underestimated TEE by approximately 9%. In 12 (63%) of 19 women, the TEE_SWA5_ was within ±5% of the TEE individually measured with the DLW method. With regard to TEE_SWA6_ and TEE_AH_, nine (47%) and seven (37%) women, respectively, were within ±5% of the TEE individually measured with the DLW method. The correlations between the criterion method (TEE_DLW_) and the TEE_SWA5_, TEE_SWA6_, and TEE_AH_ methods were all statistically significant (Fig. 1A–C).

**Table 2:** Energy expenditure measured by different methods in women with COPD (*N* = 19)

	Mean ± SD	Min–max
RMR^1^, kJ/day	4768 ± 601	3617–6248
TEE		
TEE_DLW_, kJ/day	7967 ± 1090	5318–10,258
TEE_SWA5_, kJ/day	7988 ± 945	6000–9551
TEE_SWA6_, kJ/day	7258 ± 1027	5012–9770
TEE_AH_, kJ/day	7260 ± 1048	5630–10,022
AEE		
AEE_DLW_^2^, kJ/day	3199 ± 693	1701–4594
AEE_SWA5_^3^, kJ/day	2814 ± 810	1495–4508
AEE_SWA6_^3^, kJ/day	2085 ± 810	507–3371
AEE_AH_^4^, kJ/day	2070 ± 709	827–3500

RMR, resting metabolic rate measured with indirect calorimetry; TEE, total daily energy expenditure, assessed using the DLW (TEE_DLW_), SenseWear Armband 5.1 (TEE_SWA5_) and 6.1 (TEE_SWA6_), and ActiHeart (TEE_AH_) methods; AEE, activity energy expenditure assessed using the DLW (AEE_DLW_), SenseWear Armband 5.1 (AEE_SWA5_) and 6.1 (AEE_SWA6_), and ActiHeart (AEE_AH_) methods.

1 Data represent the average of the last 25 min of measurement.

2 AEE_DLW_ = TEE_DLW_ − RMR.

3 AEE_SWA_ = TEE_SWA_ − RMR (using the Harris–Benedict equation).

4 AEE_AH_ = TEE_AH_ − AEE (with the AEE values estimated by AH).

**Figure 2:**
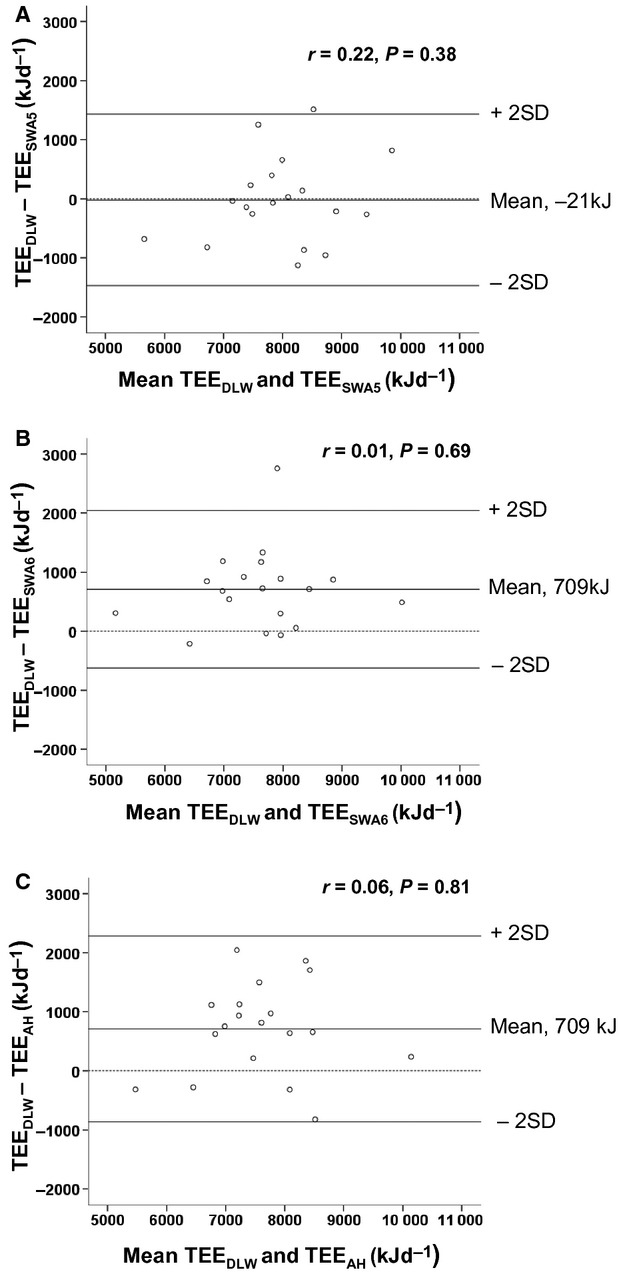
Bland–Altman plots showing the differences in the mean TEE between the DLW method (TEE_DLW_) and (A) the SenseWear Armband, version 5.1 (TEE_SWA5_), (B) the SenseWear Armband, version 6.1 (TEE_SWA6_), and (C) the ActiHeart (TEE_AH_) monitor in 19 women with COPD. TEE, total energy expenditure; DLW, doubly labeled water; COPD, chronic obstructive pulmonary disease.

Table 3 shows the agreement between the criterion method and the SWA 5.1, SWA 6.1, and AH methods with regard to the estimates of TEE as analyzed by ICC. For TEE_SWA5_, 76% of the total variance was explained by differences between the patients, whereas the other 24% of the variance was due to within-subject variation between methods. For TEE_SWA6_ and TEE_AH_, 66 and 61% of the total variance, respectively, was explained by differences between the patients. Bland–Altman plots (Fig. 2A–C) for TEE as estimated by SWA 5.1, SWA 6.1, and AH revealed that the values were evenly distributed around the mean. No systematic bias was present in these plots, indicating that no significant relationship exists between the magnitude of energy expenditure and the differences in energy expenditure between the two methods.

**Table 3:** Intraclass correlation coefficients for total and activity energy expenditure for the criterion and test methods in women with COPD (*N* = 19)

Energy expenditure	ICC	95% CI
TEE		
Criterion (DLW)		
SWA5	0.76	0.47–0.90
SWA6	0.66	0.02–0.88
AH	0.61	0.05–0.85
AEE		
Criterion (DLW and IC)		
SWA5	0.53	0.18–0.79
SWA6	0.31	−0.10 to 0.69
AH	0.29	−0.09 to 0.67

ICC, intraclass correlation coefficient; TEE, total energy expenditure; DLW, doubly labeled water; SWA5, SenseWear Armband software version 5.1; SWA6, SenseWear Armband software version 6.1; AH, ActiHeart; AEE, activity energy expenditure; IC, indirect calorimetry.

### Activity energy expenditure

#### Method 1

**Figure 3:**
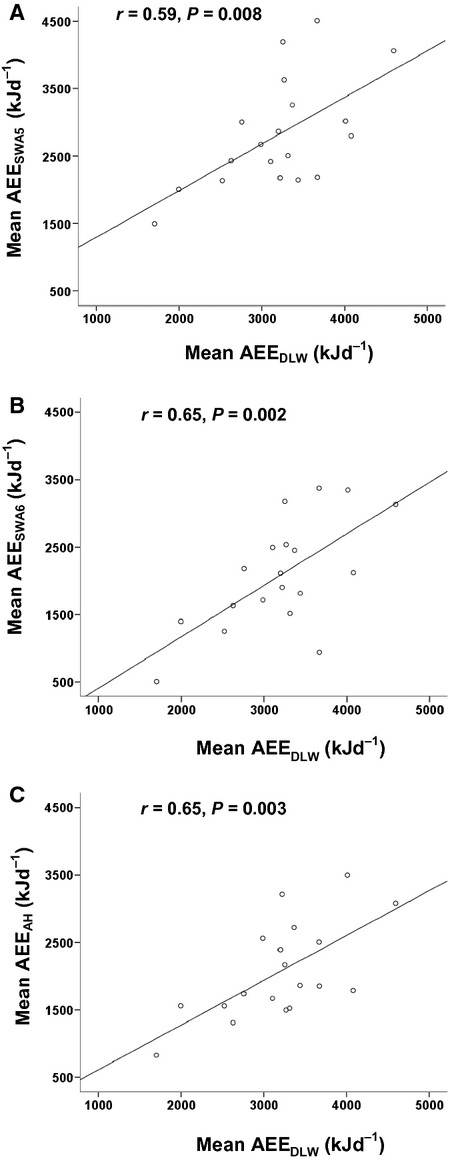
Pearson's correlations between the mean AEE measured by the DLW method (AEE_DLW_) and that measured by (A) the SenseWear Armband, version 5.1 (AEE_SWA5_), (B) the SenseWear Armband, version 6.1 (AEE_SWA6_), and (C) the ActiHeart (AEE_AH_) method in 19 women with COPD. AEE, activity energy expenditure; DLW, doubly labeled water; COPD, chronic obstructive pulmonary disease.

Table 2 shows AEE estimates obtained from the DLW, SWA, and AH methods. As with TEE, considerable variation occurred in the AEE estimates within this COPD population as measured by the DLW, SWA 5.1, SWA 6.1, and AH methods. Paired *t*-tests revealed a mean difference ± SD of 385 ± 686 kJ/day (*P* = 0.03) between AEE_DLW_ and AEE_SWA5_, 1114 ± 634 kJ/day (*P* < 0.001) between AEE_DLW_ and AEE_SWA6_, and 1128 ± 586 kJ/day (*P* < 0.001) between AEE_DLW_ and AEE_AH_. Compared with the criterion method, AEE assessments using SWA 5.1 underestimated AEE values by approximately 12%, whereas the SWA 6.1 and AH methods underestimated AEE values by approximately 35%. The correlations between the criterion method (AEE_DLW_) and the SWA 5.1, SWA 6.1, and AH methods for AEE estimates were statistically significant (Fig. 3A–C).

**Figure 4:**
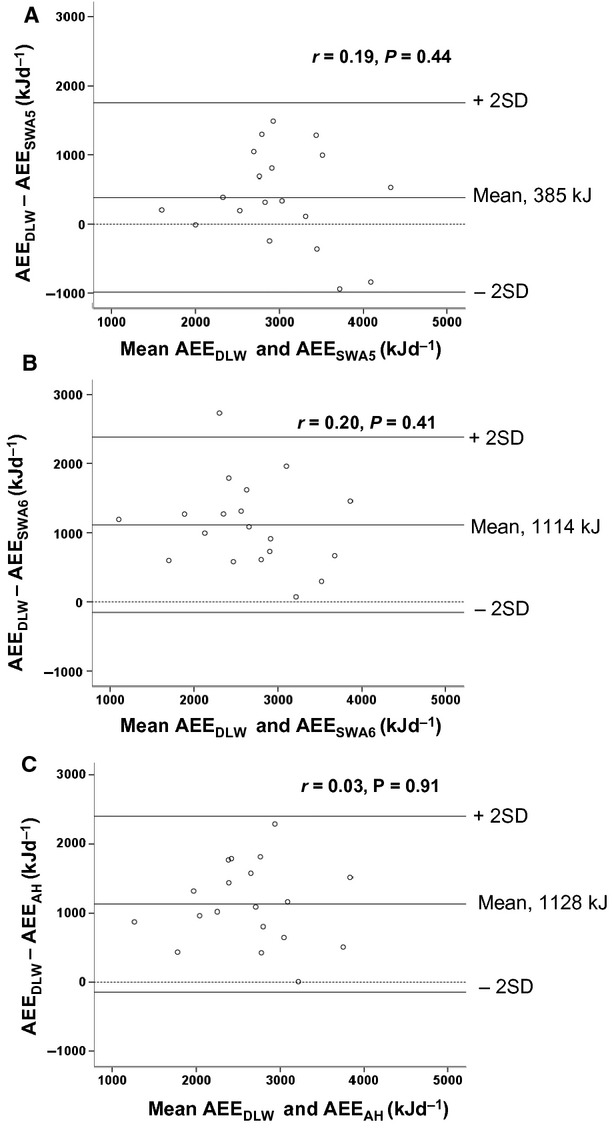
Bland–Altman plots showing the differences in the mean AEE between the DLW method (AEE_DLW_) and (A) the SenseWear Armband, version 5.1 (AEE_SWA5_), (B) the SenseWear Armband, version 6.1 (AEE_SWA6_), and (C) the ActiHeart (AEE_AH_) monitor in 19 women with COPD. AEE, activity energy expenditure; DLW, doubly labeled water; COPD, chronic obstructive pulmonary disease.

Table 3 shows the agreement between the criterion method and the SWA 5.1, SWA 6.1, and AH methods on AEE estimates, as analyzed by ICC. For AEE_SWA5_, 53% of the total variance was explained by differences between the patients, whereas 47% was due to within-subject variation between methods. For AEE_SWA6_ and AEE_AH_, 31 and 26% of the total variance, respectively, was explained by differences between the patients. In Bland–Altman plots (Fig. 4A–C) for AEE, as estimated by SWA 5.1, SWA 6.1, and AH, no significant relationship was observed between the magnitude of energy expenditure and the difference in energy expenditure between the two methods.

Substantial variation was also observed in PALs, which were calculated as the ratio between the criterion method for TEE and RMR (PAL = TEE_DLW_/RMR). The mean ± SD of the PAL was 1.67 ± 0.15(*P* < 0.0001) and ranged from 1.47 to 2.09.

#### Method 2

Table 4 shows the estimates of AEE from the criterion and test methods using RMR measured by indirect calorimetry in all the calculations of AEE. There was a large variation in AEE estimated by the DLW (1701–4594 kJ/day), SWA5.1 (1852–4806 kJ/day), SWA6.1 (913–3880 kJ/day), and AH (1058–4040 kJ/day) methods. The difference of the means between the criterion method and test methods for assessment of AEE was similar to that of TEE. The ICC analysis showed a better agreement between the criterion method and the test methods using measured RMR by indirect calorimetry in all the analysis of AEE than using estimated RMR by Harris–Benedict equation for AEE calculations by SWA.

**Table 4:** Activity energy expenditure estimates from the criterion and test methods using resting metabolic rate measured by indirect calorimetry in all the calculations of activity energy expenditure in women with COPD (*N* = 19)

	Mean ± SD	Difference of the means ± SD		Pearson's correlation		Intraclass correlations coefficient	
				
	kJ/day	kJ/day	*P*-values	*r*	*P*-values	ICC	95% CI
AEE							
Criterion (TEE_DLW_ − RMR)	3199 ± 693						
SWA5	3220 ± 791	−21 ± 726	0.90	0.71	0.01	0.70	0.20 to 0.89
SWA6	2490 ± 783	709 ± 667	<0.0001	0.75	0.005	0.59	−0.18 to 0.85
AH	2490 ± 829	709 ± 786	0.001	0.65	0.003	0.55	−0.18 to 0.80

COPD, chronic obstructive pulmonary disease; ICC, intraclass correlation coefficient; AEE, activity energy expenditure; TEE, total energy expenditure; RMR, resting metabolic rates; SWA5, SenseWear Armband software version 5.1; AH, ActiHeart.

## Discussion

The results of this validation study show that the SWA method with software version 5.1 assessed TEE with great accuracy over a 14-day period in free-living women with COPD. However, the SWA with software version 6.1 and AH methods both tend to underestimate TEE.

The agreement between AEE estimates that were obtained using the DLW and SWA with software version 5.1 methods was modest. However, the agreements between AEE estimates using the DLW and SWA with software version 6.1 methods or the AH monitor were weaker. Both the SWA and AH monitors underestimated the AEE in female COPD patients.

This study is unique in that it is the first study to assess energy expenditure exclusively in women with COPD. The use of DLW as a criterion method has been reported in several studies measuring energy expenditure in COPD populations of both genders, but it has not been used in studies specifically examining women with COPD (Baarends et al. 1997; Slinde et al. 2003). This study used DLW as a criterion method to validate SWA and AH in free-living women with COPD. In addition, we also validated the utility of the AH monitor in assessments of TEE and AEE, which had not been previously established in patients with COPD. One limitation of this study is that patients with very severe COPD were not included.

### Total energy expenditure

There were no significant differences in mean TEE from SWA5 versus DLW. There was a strong correlation between the estimates of TEE_SWA5_ and TEE_DLW_. The ICC analysis showed a strong agreement between measures of TEE_SWA5_ and TEE_DLW_, and Bland–Altman plots revealed no systematic bias. The difference in mean TEE from SWA6 and AH versus DLW was significant. There was a strong correlation between the estimates of TEE_SWA5_ and TEE_DLW_. The ICC analysis showed a good agreement between measures of TEE_SWA6_, respectively, AH and TEE_DLW_, and Bland–Altman plots revealed no systematic bias.

Although no validation study like ours has been conducted previously, the results of this study are consistent with previous reports that have validated SWAs in the estimation of TEE in other adult populations (St-Onge et al. 2007; Johannsen et al. 2010; Mackey et al. 2011). Rabinovich et al. (2013) have an exploratory analysis and compared different physical activity monitors (including SenseWear Armband [SWA]) that report TEE as outcomes. The results varied between the different monitors. The SWA they used in their study was a newer version with triaxial accelerometer, whereas the SWA in the present study was biaxial. Johannsen and colleagues used SWA software version 6.1, St-Onge et al. (2007) used version 4.02, and Mackey et al. (2011) validated both versions 5.1 and 6.1. In the present study, SWA software version 5.1 performed better than version 6.1, a finding that has been reported elsewhere (Mackey et al. 2011). SWA has been previously validated in laboratory settings for TEE estimations in COPD patients using criterion methods other than the DLW method, and our results are in concordance with these studies (Patel et al. 2007; Langer et al. 2009; Hill et al. 2010; Cavalheri et al. 2011). Hill et al. (2010) showed that estimations of TEE using SWA are sensitive to small but important changes. Based on the results of the present study and other studies (Bäcklund et al. 2010; Mackey et al. 2011), it is important to choose a reliable software version, such as SWA software version 5.1, which provides more accurate estimates of TEE than software version 6.1. Because the algorithms in software versions 5.1 and 6.1 are proprietary, it is not possible to speculate why version 5.1 outperforms version 6.1.

We conducted further analyses based on the primary results of TEE (assessed by SWA 5.1) to reliably assess the minimum number of days required to measure TEE. These analyses indicated that at least 4 days of measurement are required to reliably assess TEE (data not shown).

The estimates of TEE using AH monitors exhibited moderate agreement with the results obtained using the criterion method, although AH did lead to underestimations of approximately 9%. To date, no validation studies have been conducted involving adults with chronic diseases. In contrast to other studies that have used modified adapted multivariate regression models, we used the manufacturer's algorithms in our study (Butte et al. 2010; Zakeri et al. 2010). AH requires an 8-min step test to synchronize the device at an individual level. None of the patients in the current study were able to complete the whole step test due to various reasons, including breathlessness, which was the most common important factor. It remains unclear whether this lack of test completion had any impact on the TEE estimations in the current study. Similarly, whether the study outcomes would have been affected if the device had been synchronized at a group level is not known. Because breathlessness is a major symptom of COPD, synchronizing AHs at a group level may be recommended. Additional studies comparing the effects of synchronizing AHs at the individual and group levels in populations with chronic disease are required.

### Activity energy expenditure

#### Method 1

The estimation of AEE with SWAs was less precise than the estimation of TEE. The primary strength of the SWA is that it combines multiple physiological heat-related factors with motion data from a biaxial accelerometer, which assess energy expenditure even during nonambulatory and low-intensity activities that are common among this patient population.

The strength of AH device is that it allows physical activity to be recorded synchronously with heart rate. This allows the AH to accurately measure AEE even when the exercise has low body movement (but high heart rate) with exercise such as static cycling that may be otherwise missed by an accelerometer alone. It also prevents false readings when stress or stimulation cause the heart rate to rise, or when low-level vibrations are picked up by the accelerometer that could be mistaken for “steps” or exercise.

The results of the present study suggest that the SWA version 5.1 assesses AEE more accurately than version 6.1 in women with COPD. These results are somewhat consistent with other studies conducted on adults of all ages (St-Onge et al. 2007; Mackey et al. 2011; Rabinovich et al. 2013). Rabinovich et al. (2013) have recently compared the validity of different physical activity monitors including SWA with regard to their ability to measure AEE, to capture small changes in physical activity and user friendliness. The results varied among the monitors. The SWA they used had a triaxial accelerometer, whereas in our study, it was a biaxial accelerometer. They have used METs and steps for the assessment of PAL, whereas in this study, the validity of assessed AEE was studied. Because SWA software does not provide estimates of RMR, we calculated AEE using estimates of TEE that were obtained using the SWA, and we used the Harris–Benedict equation for women to estimate RMR (Mackey et al. 2011). It is possible that this method influenced the outcome of AEE estimations obtained using SWAs.

Although the criterion method and the AH method were somewhat similar with regard to their AEE assessments, the AH method underestimated AEE by approximately 35%. Despite the fact that some reports have shown AHs to provide a reliable assessment of AEE in children and adult men (De Bock et al. 2010; Takken et al. 2010; Villars et al. 2012), the results of the present study cannot be compared with those studies because our study group consisted of adult women with a chronic disease.

#### Method 2

The estimates of AEE from SWA and AH using measured RMR from indirect calorimetry with regard to difference of the means, Pearson's correlation, and ICC analysis were almost similar to that of total energy expenditure. This could be because RMR value from indirect calorimetry is then a common denominator for all calculations of TEE and AEE. Using measured RMR by indirect calorimetry in all the calculations of AEE showed better agreement between the criterion and the test methods.

There are a couple of reasons why we used RMR by Harris–Benedict equation to calculate AEE from SWA. Firstly, as mentioned above, SWA does not assess RMR. In clinical settings, the availability to indirect calorimetry is very limited. The purpose of the present study was to have reliable objective methods which can be used “off the shelf” in the clinical settings. Secondly, AH gives an assessment of RMR and to use it in calculating AEE by AH seems logical, rather than replacing it with measured RMR by indirect calorimetry.

In general, the study participants tolerated SWAs and AHs well over the entire study period. No reports were received related to technical problems or usage discomfort for these monitors. Several participants reported being more comfortable with the SWA, but others preferred the AH. The SWA is simple to administer, and no preparation or training prior to long-term registration is required. To initialize the SWA, patient characteristics, such as age, gender, weight, height, smoking status, and handedness, are used. The AH monitor, however, requires a signal test in addition to these patient characteristics to ensure the correct placement of the device and a step test to synchronize the AH at an individual level prior to long-term registration. These requirements are more time consuming and demanding for some patient groups, such as COPD patients. The patients in our study reported that they found the step test to be very demanding, and they were not able to complete the entire test.

In conclusion, the results of this validation study show that the SWA version 5.1 reliably estimates TEE in free-living women with COPD. However, the SWA 6.1 and AH monitors tend to underestimate TEE. Assessments of AEE using the SWA and AH monitor were less accurate compared with those obtained using the DLW method. Future studies examining patients with all stages of COPD, especially patients with very severe COPD, are required because patients with severe forms of COPD have been shown to have the most problems with regard to symptoms, comorbidities, and nutritional status.
